# Wearable Triboelectric Nanogenerators Based on Polyamide Composites Doped with 2D Graphitic Carbon Nitride

**DOI:** 10.3390/polym14153029

**Published:** 2022-07-26

**Authors:** Yana Xiao, Bingang Xu, Qi Bao, Yintung Lam

**Affiliations:** Nanotechnology Center, Institute of Textiles and Clothing, The Hong Kong Polytechnic University, Hung Hom, Kowloon, Hong Kong 999077, China; yana01.xiao@connect.polyu.hk (Y.X.); pauki.bao@polyu.edu.hk (Q.B.); yt-oskar.lam@connect.polyu.hk (Y.L.)

**Keywords:** triboelectric nanogenerator, energy harvesting, electrospinning, two-dimensional material, graphitic carbon nitride

## Abstract

Triboelectric nanogenerators (TENGs) have attracted many researchers’ attention with their remarkable potential despite the fact that the practical implementation requires further improvement in their electric performance. In this work, a novel graphene phase two-dimension material, graphitic carbon nitride (g-C_3_N_4_), was employed for the development of a TENG material with enhanced features. An electrospun nanofibrous PA_66_ membrane doped with g-C_3_N_4_ was fabricated as a multifunctional TENG for harvesting different kinds of mechanical energy and detecting human motions. By utilizing the innovative 2D material in PA_66_ solution for electrospinning, the as-made TENG showed a two times enhancement in electrical performance as compared to the control device, and also had the advantages of lightweight, softness, high porosity, and rugged interface properties. The assembled TENG with 4 cm^2^ could light up 40 light-emitting diodes by gentle hand clapping and power electronic watches or calculators with charging capacitors. At a given impact force of 40 N and 3 Hz, the as-made TENG can generate an open-circuit voltage of 80 V, short current of ±3 µA, charge transfer of 50 nC, and a maximum power density of 45 mW/m^2^ at a load resistance of 500 MΩ. The UV light sensitivity of TENG was also improved via g-C_3_N_4_ doping, showing that charge transfer is very sensitive with a two times enhancement with dopant. For the demonstration of applications, the g-C_3_N_4_ doped TENG was fabricated into an energy flag to scavenge wind energy and sensor devices for detecting human motions.

## 1. Introduction

Burgeoning concerns over the energy crisis and zero carbon emissions stimulate research on various new energy harvesting methods to replace traditional energy resources such as fossil fuels, among which triboelectric nanogenerators (TENGs) have become an efficient, versatile, and attractive candidate. TENGs are normally composed of two dissimilar materials with different electron affinities, and their mechanism involves the physical principle of triboelectrification and electrostatic induction coupling as well as the Maxwell displacement current. However, the development of TENGs has encountered some bottlenecks such as low electrical performance, low energy efficiency, as well as the limited application environment. Recently, much research endeavor has been committed to the enhancement of the TENG’s output performance. One major stream is to design and fabricate micro or nano architectures using ion etching processes [1], such as photolithography, nanoimprinting lithography, and laser interference. Another approach is to improve the friction composites’ permittivity through doping certain materials into pristine triboelectric layers [2], such as carbon nanotubes, liquid metals, graphene oxide, metal nanowire/nanoparticle, and BaTiO_3_. Single-layer materials [3] or two-dimensional (2D) nanomaterials are generally categorized into either 2D allotropes or compounds with crystalline solids consisting of a single layer of atoms. It is of note that various 2D structures [4] have been discovered, such as graphene, MXene, MoS_2_, WS_2_, and graphitic carbon nitride (g-C_3_N_4_). Graphitic carbon nitride (g-C_3_N_4_) has demonstrated salient and promising features in applications such as hydrogen evolution, photocatalytic degradation, gas sensors, antibacterial structures, and microwave absorbers [5]. For instance, pure g-C_3_N_4_ nanosheets show attractive functions owing to their photo absorption characteristics, bio-friendly nature, mechanical flexibility semiconductor properties [6], etc. Their appropriate energy gap level [7] and the favorable positions of valence bands facilitate the generation of electron–hole pairs and promote the deposition of electrons under light irradiation. Meanwhile, g-C_3_N_4_ possesses the merits of facile synthesis, high stability, and good biosafety, as compared to other competitors such as TiO_2_. Hence, it is highly expected that novel and multi-functional TENGs could be obtained if 2D g-C_3_N_4_ can be properly designed and integrated into the devices. However, according to our best knowledge, there is no such work in incorporating 2D g-C_3_N_4_ into electrospinning TENGs so far [8].

In practice, the majority of the doping work has been focused on the electronegative dielectric medium, such as polydimethylsiloxane (PDMS), whose pristine phase is liquid under normal circumstances. Compared with various preparation methods, electrospinning is a versatile, simple, economic, and accessible way [9] to produce ultrathin fiber membranes with the necessary length, sufficient surface area, and comparatively intrinsic porosity. In past research, electrospinning has been adopted as a suitable method in the field of energy harvesting for fabricating diverse nanofibrous surface microstructures [10] from economical commercialized polymer materials, such as nylon (polyamide) [11], polyvinylidene fluoride (PVDF), polytetrafluoroethylene (PTFE), and ion gel. Moreover, nanofiber membranes made from electrospinning have featured the advantages of lightweight, softness, and high porosity, which are superior to the dense films or fabricated textiles with micron-sized pores for the production of wearable TENGs. As inspired by TiO_2_, the addition of g-C_3_N_4_ might also produce an effective self-cleaning effect and the recovery of device performance [12]. The addition of g-C_3_N_4_ in PA_66_ could improve the electrical power output efficiency of a TENG under light, and it is better to enable a built-in light sensitive function as a wearable self-powered sensor. In addition to experimental work, theoretical studies on Maxwell equations, capacitor model, mechanical model frequency, quantum model for charge transfer, triboelectric charge density (TECD), and finite-element modeling simulations have also attracted many researchers’ attention [13,14,15,16,17,18].

Herein, for the first time we adopted semiconductor g-C_3_N_4_ into the electrospinning membrane PA_66_ to assemble a multifunctional TENG which could harvest different kinds of mechanical energy and detect human motions. By adopting the innovative 2D material, we doubled the electric performance of the TENG with the micro-architectured composition of electropositive dielectric polyamide. The TENGs prepared with the electrospinning membrane could be used as flexible power-generating devices to scavenge the kinetic energy of human motion or as flags to capture wind energy. The assembled TENG with a 4 cm^2^ PA_66_ electrospinning membrane with doping g-C_3_N_4_ nanosheets can achieve more than 80 V of voltage and 45 mW/m^2^ of power density, which was capable of lighting up 40 light-emitting diodes by gentle hand clapping. Moreover, a photoactive substance causing different performances of the TENG with the absence and presence of UV light was first attempted. Our research could provide a useful approach for the fabrication and modification of wearable TENGs with customizable functionalities for different applications. 

## 2. Materials and Methods

### 2.1. Materials

Melamine (C_3_H_6_N_6_, 99% purity), acetic acid, N, N-dimethylformamide (DMF, ≥99.8%, ACS reagent), formic acid (88−91%, ACS reagent), tetrahydrofuran (≥99.9%, ACS reagent), acetone (≥99.5%, ACS reagent), ethanol, PVDF, polyacrylonitrile (PAN), and polyimide (PI) were products of DIECKMANN. PDMS (SYLGARD 184 Silicone Elastomer kit) was purchased from Dow Corning Co, Ltd. Polyamide (PA_66_) staple fiber was purchased from Shandong Zhongxian Textile Technology Co, Ltd., China. Copper (Cu) wire and the aluminum foil were purchased from Dongguan Yishengxing Copper and Aluminum Materials Co, Ltd., Dongguan, Guangdong, China. Cu/Ni coated fabric applied as the conductive fabric was purchased from 3M Corp. All reagents were used as received without further purification. 

### 2.2. Synthesis of g-C_3_N_4_

The g-C_3_N_4_ powders were synthesized by the direct pyrolysis method. In brief, the melamine was applied as a raw material and pure melamine (C_3_H_6_N_6_, 99% purity) powder was firstly heated in a quartz tube furnace up to 600 °C from room temperature at a heating rate of 5 °C per minute. The heating was maintained for over 8 hrs in a N_2_ protective atmosphere. Once the furnace was cooled down to room temperature naturally, the as-synthesized powders were ground and rinsed with deionized water, then they were filtered and squeezed in a mortar to obtain comparatively pure g-C_3_N_4_, and the product was finally dried in a vacuum drier under 50 °C overnight. To minimize the effect of agglomeration of g-C_3_N_4_ particles for subsequent doping, g-C_3_N_4_ powders were first crushed in a mortar, put into a small amount of alcohol, and then dispersed under ultrasonic vibration for more than six hours before usage, and then the alcohol solvent was removed by evaporation to make the powder as small as possible to be dispersed into a polymer solution [9,19,20,21]. Since g-C_3_N_4_ was indiscerptible in the solvent of formic acid and slightly soluble in water or a common organic solvent such as ethanol, the suspension solution of g-C_3_N_4_ increased the load capacity and made electrospinning less stable, which indirectly led to the agglomeration of g-C_3_N_4_ particles. Therefore, the maximum loading capacity of g-C_3_N_4_ in electrospinning membranes was limited to 0.7 wt% according to the result of our experiments.

### 2.3. Fabrication of the PA_66_ Membrane

To further tackle the aforementioned insolubility problem, a mixture of solvents (50% formic acid and 50% acetic acid) was adopted for PA_66_ dissolution at a concentration of 15 wt%, which had suitable volatility and little pungent smell. The g-C_3_N_4_ powder was mixed into the electrospinning solution according to different concentrations, and the dispersions of g-C_3_N_4_ and PA_66_ were prepared by ultrasonication overnight. Then, the as-obtained solutions were used for electrospinning doped PA_66_ at the voltage of 24 kV with the drum collector at the rotating rate of 300 rpm and the liquid feed rate of 2 mL/h, with an air heater at 80 °C and a dehumidifier for solutions with the dopant. 

**Figure 1:**
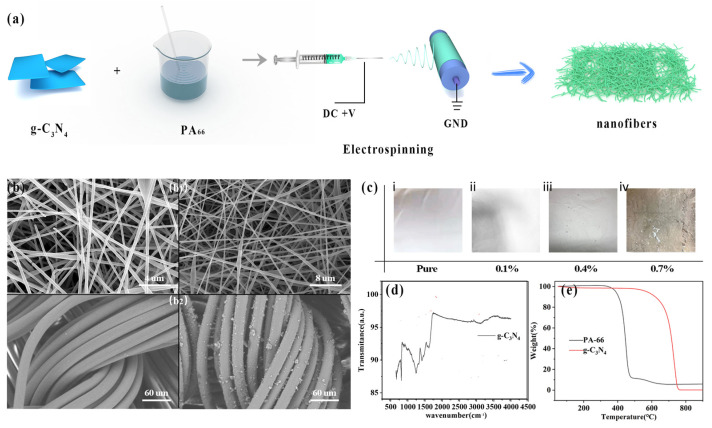
(**a**) Schematic illustration for preparation of electrospinning membrane. (**b**) SEM images of (**b_1_**) electrospinning PA_66_ and (**b_2_**) nylon textile cloth at different magnifications. (**c**) Photographic images of electrospinning membrane PA_66_ with different concentrations: (i) pure, (ii) 0.1 wt%, (iii) 0.4 wt%, and (iv) 0.7 wt% of g-C_3_N_4_. (**d**) FTIR spectrum of absorption spectra for g-C_3_N_4_ powders. (**e**) TGA plots of PA_66_ and g-C_3_N_4_.

The PA_66_ electrospinning membranes with 0.1 wt% of g-C_3_N_4_ (Figure 1c(ii)) and 0.4 wt% of g-C_3_N_4_ (Figure 1c(iii)), as well as the pure PA_66_ membrane (Figure 1c(i)), were prepared under the same conditions, respectively. Because of the limited solvability of solid-state g-C_3_N_4_ powder, the maximum concentration in the experiments for electrospinning dispersions with dopant nanoparticles of graphitic carbon nitride was limited to 0.7 wt% (Figure 1c(iv)), and the material with 0.7 wt% g-C_3_N_4_ was also easily chapped and unsuitable for fabricating into TENG devices.

### 2.4. Material Characterization and Measurement of Electrical Output Performance

Fourier transforms infrared spectra (FT-IR) were recorded on a Spectrum 100 Perkin Elmer spectrometer. The scanning electron microscope (SEM) images were taken from a Hitachi TM-3000 Tabletop Microscope as well as a field emission scanning electron microscope TESCAN MAIA3. The cyclic contact-separation motion of TENG triboelectric performance measurements was realized by a life test machine (ZX-A03, Zhongxingda, Shenzhen, China) with a force gauge INTERFACE to quantify the impact force. The thermogravimetric analysis (TGA) curve was obtained on a Mettler Toledo TGA/DSC1 system. The output open circuit voltage was recorded by a multifunctional oscilloscope (DSOX3024T, InfiniiVision), while the output short circuit current and transfer charge were measured by an electrometer (Keithley 6514, Tektronix). The air permeability was evaluated by a KES-F8-AP1 Air Permeability Tester (KATO TECH CO., Ltd. Kyoto, Japan).

## 3. Results and Discussion

We developed g-C_3_N_4_ doped PA_66_ nanofibers using electrospinning as illustrated in Figure 1a. Figure 1b shows the SEM image of the electrospinning PA_66_ film and nylon textile, respectively. As the concentration of g-C_3_N_4_ was increased to 0.7 wt%, the electrospinning membranes had rough particles on the surface and the membrane was fragmented and unable to fabricate TENGs (Figure 1c(iv)). This kind of phenomenon has also been observed in other experiments with insoluble dopants such as liquid metal. From the Fourier transform infrared spectra (Figure 1d) of the synthesized pyrolysis, several strong bands in the 1200–1650 cm^−1^ region corresponded to typical CN heterocycles. Moreover, thermal gravimetric analysis (TGA) showed that the thermal decomposition temperature of PA_66_ was between 450 and 500 °C, and that of g-C_3_N_4_ was between 600 and 750 °C, as shown in Figure 1e. 

**Figure 2:**
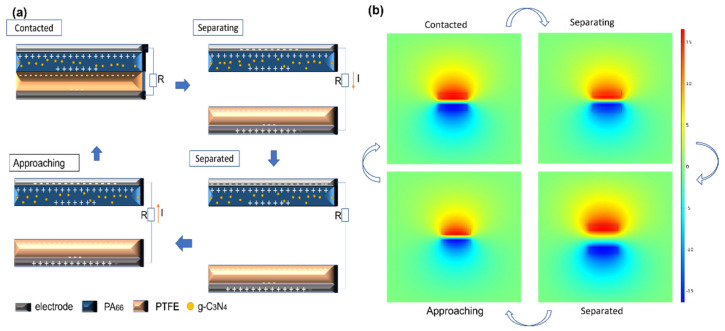
(**a**) Schematic diagrams of working mechanism for contact-separation mode electrospinning membrane TENG with g-C_3_N_4_ dopant. (**b**) Simulation results of electrical potential distribution by COMSOL software.

The electrospinning membrane TENG was assembled by adopting the common contact separation mode, using the PA membrane as the positive tribo-layer, PTFE as the negative tribo-layer, and aluminum foil and conductive copper nickel fiber as the electrode. The operating principle of the electrospinning membrane TENG is schematically illustrated in Figure 2a, exhibiting the process of electron transfer in a contact separation mode. Subject to a periodic external force, in the initial state (Figure 2a), the positive dielectric PA_66_ electrospinning membrane with or without doped g-C_3_N_4_ contacted together with the negative dielectric under external force, where the positive charges and negative charges were produced on the surface of both membranes, respectively, making no electron transfer there. In the second state (Figure 2a), when the external force separates these two membranes with a gap, an electric potential difference was produced, and then electrons were transferred through an external load (or electrometer) from one electrode to another, so an electrical current was produced. Once two membranes were separated and the gap went to the maximum (Figure 2a), the electrostatic equilibrium made no electrons move. When the external force broke the electrostatic equilibrium, a new opposite potential difference was generated, and the electron current flowed in an opposite direction (Figure 2a). During the process of contact and separation, electrons flowed between the bottom and top electrodes continuously and generated alternating current (AC). The whole power generation process and potential distribution were simulated and demonstrated by COMSOL Multiphysics software, as shown in Figure 2b. 

**Figure 3:**
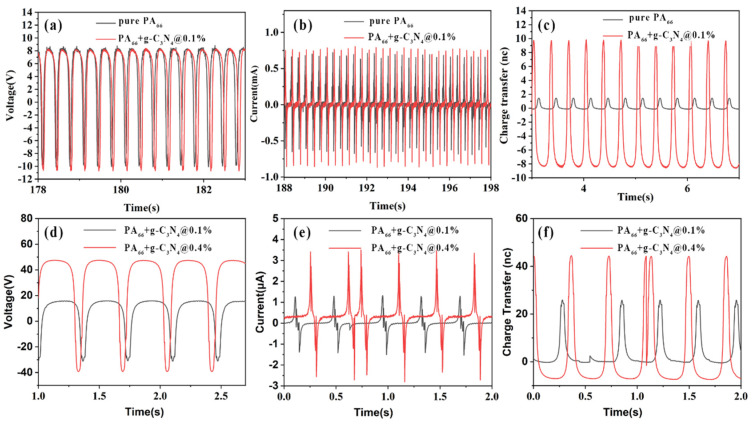
Output performance comparison of electrospinning membranes between pure PA_66_ and electrospinning membrane PA_66_ with 0.1 wt% doped g-C_3_N_4_ (low concentration): (**a**) open-circuit voltage, (**b**) short circuit current, and (**c**) charge transfer. Output performance comparison of electrospinning membrane PA_66_ with 0.1 wt% doped g-C_3_N_4_ (low concentration) and 0.4 wt% doped g-C_3_N_4_ (high concentration): (**d**) open-circuit voltage©, (**e**) short circuit current, and (**f**) charge transfer.

The output performance of the electrospinning membrane of pure PA_66_ and PA_66_ doped with g-C_3_N_4_ in vertical contact-separation mode with the negative PTFE layer was systematically studied with results shown in Figure 3. At a given impact force of 30 N and 3 Hz, with a contact area at 4 cm^2^ (2 cm × 2 cm), compared with the pure electrospinning membrane PA_66,_ the open circuit voltage of the PA_66_ doped with g-C_3_N_4_ was maintained at a similar value of around 18 V (Figure 3a). The short circuit current of the doped PA_66_ was 1.5 µA, which was 0.3 µA higher than that of the pure PA_66_ (Figure 3b). However, the transfer charge presented significant sensitivity after doping with g-C_3_N_4_ even at a very low concentration of 0.1 wt%, with about a 12 times enhancement from 1.5 nC to 18 nC (Figure 3c). By increasing the concentration from 0.1 wt% to 0.4 wt%, all three parameters—open circuit voltage, short circuit current, and transfer charge—were almost doubled, as shown in Figure 3d–f. At the concentration of 0.4 wt%, the as-made TENG showed the largest open-circuit voltage of 80 V, short current of ±3 µA, and charge transfer of 50 nC. 

**Figure 4:**
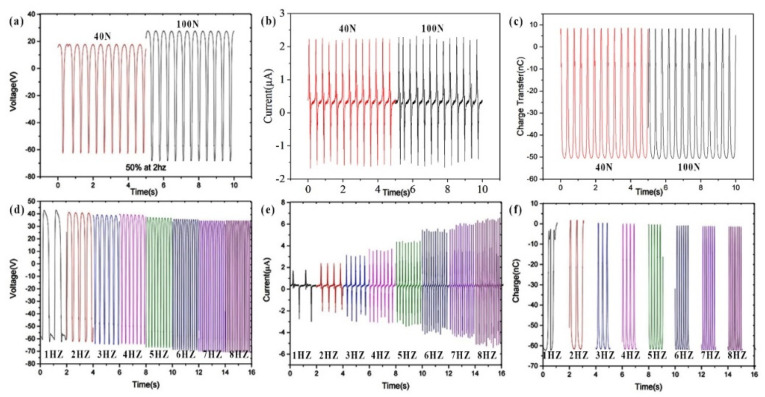
(**a**) Open-circuit voltage, (**b**) short-circuit current, and (**c**) charge transfer of doped PA_66_ electrospinning membrane TENG with different impacting forces at 40 N and 100 N intensity under the same frequency of 3 Hz. (**d**) Open-circuit voltage, (**e**) short-circuit current, and (**f**) charge transfer of PA_66_ electrospinning membrane TENG with different frequencies from 1 Hz to 8 Hz under the same impact force of 100 N.

Considering that impact forces and frequencies might vary in practical applications, the influences of doped PA_66_ TENG under different forces and frequencies were also studied here separately, as shown in Figure 4. It can be seen that the force’s impact was insignificant (Figure 4a–c) as compared to the impact of frequency, with the open circuit voltage increasing a little from 80 V to 100 V while the impact force increased from 40 N to 100 N. As for the influence of frequency (Figure 4d–f), the current increased gradually from about ±1.5 µA to ±7 µA as the frequency was increased from 1 Hz to 8 Hz (Figure 4e), while its voltage (Figure 4d) and charge transfer (Figure 4f) remained almost the same under different frequencies. One possible explanation might be that a higher impact frequency can stimulate the flow of external electrons in a shorter time under similar forces, which may result in an increased current output. The thin electrospinning membrane cannot hold a large surface charge and surface potential, and thus the change insignificantly affected the voltage. In addition, the charge transfer is already sensitive and significant even at lower frequencies, so the charge may not be able to increase significantly at higher frequencies. The power density, intrinsic impedance, and energy conversion efficiency of energy harvesting from electrospinning membrane TENGs with this material should be further optimized in the future by regulating materials composition, inventing unique structures, and employing power management circuits [22,23,24,25].

**Figure 5:**
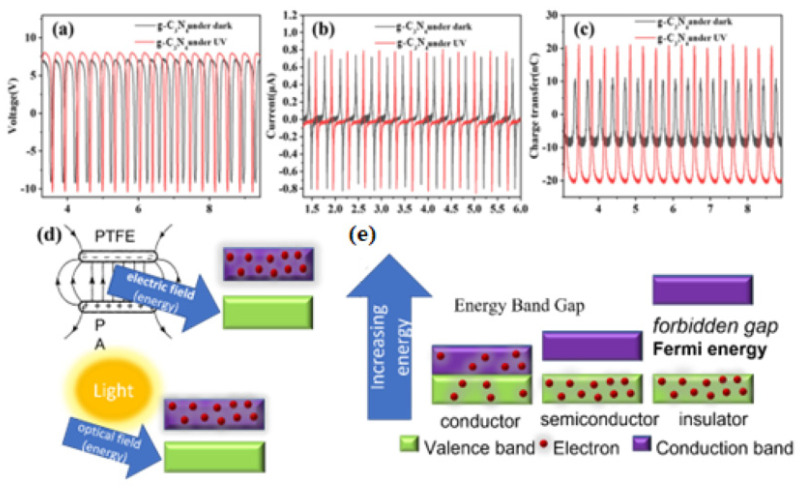
Electrical output comparison of electrospinning membrane PA_66_ TENG with doped g-C_3_N_4_ under UV light condition and dark condition: (**a**) open-circuit voltage, (**b**) short circuit current, and (**c**) charge transfer. (**d**) Illustration of electric and light field influence. (**e**) Energy bandgap theory of g-C_3_N_4_.

The change between the energy band and conduction band of a semiconductor contributes to the sensitivity of the charge transfer. As shown in Figure 5c, even 0.1 wt% low-concentration doping could still show great changes in the charge transfer (from 20 nC to around 40 nC) under ultraviolet light conditions as compared to dark conditions, but it has little effect on the voltage and current (Figure 5a,b). g-C_3_N_4_ is a light-sensitive semiconductor, and its rich nitrogen element is susceptible to losing electrons, so adding it to the positive electrode dielectric can produce a superimposed triboelectrification generation effect. The C-N atom in its structure is sp2 hybridized to form a highly delocalized π-conjugated system. Among them, the Np_z_ orbital constitutes the highest occupied molecular orbital (HOMO) of g-C_3_N_4_, and the Cp_z_ orbital constitutes the lowest unoccupied molecular orbital (LUMO). Its bandgap is around 2.7 eV, which can absorb blue-violet light with a wavelength of less than 475 nm in the solar spectrum. Under light conditions (365 nm UV), the electrons in g-C_3_N_4_ can be easily lost, because the light is also one kind of electromagnetic wave, as illustrated in Figure 5d. Charge-transferring between the discretized energy levels generates energy band gaps. The intermolecular interactions facilitate the electron hopping, developing microcurrents, and conductive networks (Figure 5e). The charge circuits in the produced conductive loops develop secondary fields and induce the magnetic moments in the 2D structures [5,26,27,28,29].

The magnetic and electric fields’ coupling can enhance each other macroscopically from microscopically. Therefore, the g-C_3_N_4_ material used in the positive electrode is much more effective than the negative electrode. As a comparison, we also conducted a comparative experiment of PVDF doped g-C_3_N_4_, which used the electrospinning membrane of PVDF with and without doping g-C_3_N_4_ powder as the negative dielectric while utilizing the PA_66_ electrospinning membrane as the positive dielectric, and the preparation and measurement of the TENG were the same as the above (using the contact separation mode and Cu/Ni coated conductive fabric as an electrode), where we found that there were slight differences in the open-circuit voltage, short circuit current, and charge transfer of the PVDF membrane with and without doping g-C_3_N_4_ (Figure S2 in the Supporting Information), which is consistent with our expectation. For comparison, a polyacrylonitrile (PAN) electrospinning membrane TENG was also fabricated and evaluated, where the short circuit current was also increased generally from about ±1 µA to ±4 µA at frequencies of 2 Hz to 10 Hz (Figure S1b), while the open circuit voltage was increased slightly from 35 V to 60 V at frequencies of 2 Hz to 6 Hz and then kept almost constant afterward (Figure S1a). Moreover, the electrospinning membrane surpassed most of the other films employed in other TENG applications, and this could be attributed to its nano-scale high porosity (Figure S4 in the Supporting Information). This feature also offers advantages such as degradability (Figure S3 in the Supporting Information), as well as disadvantages such as vulnerability, as shown in Figure S5 of the Supporting Information. FESEM of different magnifications for the electrospinning fiber and g-C_3_N_4_ powder is also shown in Figure S7 in the Supporting Information.

**Figure 6:**
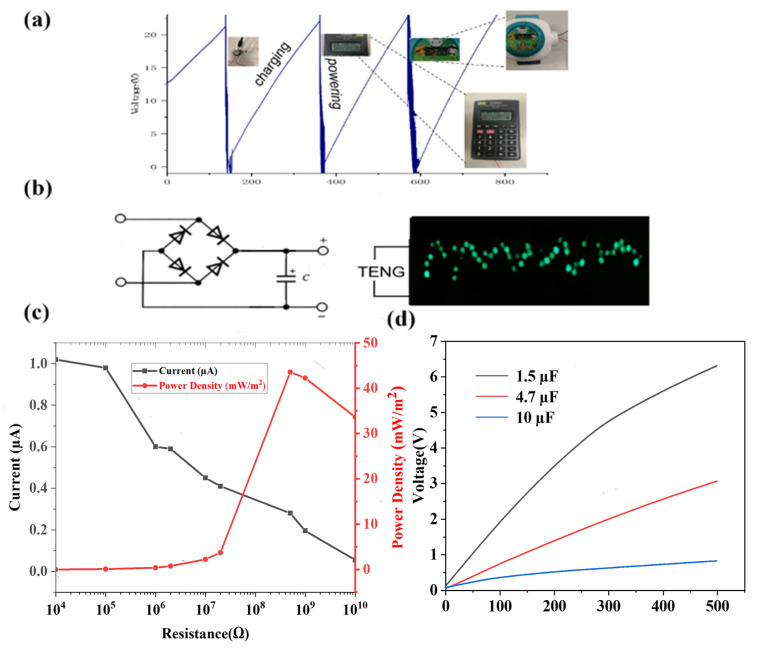
(**a**) Charging voltage curves of the electrospinning membrane TENG in the process of continuous powering devices such as calculators and electronic watches. (**b**) Illustration of electrospinning membrane TENG for lighting up 40 LEDs. (**c**) Dependence of output current and power density of the electrospinning membrane TENG on load resistances. (**d**) Charging curves of 1.5 µF, 4.7 µF, and 10 µF capacitors charged by the electrospinning membrane TENG.

The charging capacity of the doped electrospinning membrane was also evaluated by using different capacitors, with results shown in Figure 6d. The charging rates of the different capacitors were calculated as 14 mV/s for 1.5 μF, 6 mV/s for 4.7 μF, and 2 mV/s for 10 μF, respectively. As a demonstration, the continuously generated energy was used to directly drive an electronic watch and calculator. The output power density of the electrospinning membrane was also systematically evaluated by connecting with different external load resistances. At a fixed frequency of 3 Hz and impact force of 40 N, different resistors from 10 kΩ to 10 GΩ were externally connected to measure the output current, where the output current decreased with the increase of load resistance. Based on the measurements, the power density was calculated and reached the maximum value of 45 mW/m^2^ at a load resistance of 500 MΩ (Figure 6c). The output power generated by the electrospinning membrane TENG can light up at least 40 LEDs connected in series by gentle hand clapping (Figure 6b and Video S1 in Supporting Information) and can also power an electronic watch and calculator by the capacitors it charges (Figure 6a) [30,31,32].

**Figure 7:**
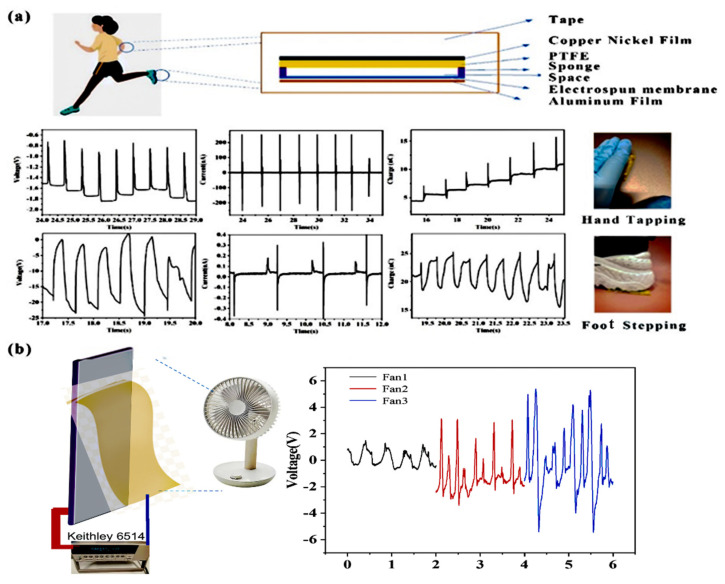
(**a**) Application of electrospinning membrane TENG for harvesting human motions and (**b**) electrospinning membrane TENG for scavenging wind energy under different levels of fan speed.

Owing to its good flexibility, the electrospinning membrane has potential applications in energy harvesting and biomotion sensing. In order to study the electric performance, the doped electrospinning membrane was also assembled into an energy device and fixed at different positions of the human body to detect biomechanical energy from human motions. The structure of this wearable energy device, as illustrated in Figure 7a, where our electrospinning membrane acted as the positive tribo-layer, was adhered to an aluminum film electrode, while PTFE as the negative tribo-layer was adhered to a Cu/Ni conductive fabric electrode, and then they were assembled using sponge for separation and finally wrapped with insulating tape outside. As a potential material for wearable device energy suppliers, the air resistance property was also tested and is shown in Figure S6 of the Supporting Information. It was found that the resistance of the electrospun membranes was only slightly higher than that of the nylon fabric, indicating the air permeability of the electrospun membranes was comparable to that of apparel fabric. This thin energy device can be fixed on shoes or gloves, generating electricity during human motion, which also showed the potential application as a wearable electronic signal sensor (Figure 7a). For example, gentle tapping by the hand can generate an electric pulse of 1 V, ±200 nA, 5 nC, and the output of stepping voltage, current, and charge transfer with the foot were around 20 V, ±0.4 µA, and 10 nC, respectively. Meanwhile, due to the light sensitivity of g-C_3_N_4_, the increase of the electric signal such as voltage could also reflect the UV information outdoors with transparent epoxy/PMMA resin packaging.

For superior flexibility, the doped electrospinning membrane could enable itself to be fabricated into a flag to scavenge wind kinetic energy (Figure 7b). With a similar structure, PTFE was adhered to conductive film and fixed to a vertical board; the electrospinning membrane was adhered to soft conductive fabric and the upper side was fixed and placed near the PTFE board. The fan can generate three levels of different wind speeds (Fan1 with a low wind speed of 2.1 m/s, Fan2 with a middle wind speed of 3.0 m/s, and Fan3 with a high wind speed of 3.9 m/s). When the wind blew at the electrospinning membrane, making the membrane contact and separate from the PTFE film intermittently, this thin TENG was able to generate electricity with an output positively related to wind speed, that is, 3 V, 6 V, and 9 V at 2.1 m/s, 3.0 m/s, and 3.9 m/s, respectively.

## 4. Conclusions

In summary, we have designed and fabricated a novel and multifunctional TENG with PA66 nanofibers doped with 2D g-C_3_N_4_. Resultantly, the doped TENG at 0.4 wt% showed twice the electrical output performance as compared to the pure PA_66_ electrospinning membrane. The addition of g-C_3_N_4_ makes a tremendous difference with the charge transfer even at 0.1 wt% concentration. The presence of light, low-concentration doping of g-C_3_N_4_ could also increase the charge transfer as a result of photogenerated electron–hole pairs. The charging rates of different capacitors were calculated as 14 mV/s for 1.5 μF, 6 mV/s for 4.7 μF, and 2 mV/s for 10 μF, respectively. The assembled TENG with 4 cm^2^ could light up 40 light-emitting diodes by gentle hand clapping and power electronic watches or calculators with charging capacitors. The as-made TENG can generate an open-circuit voltage of 80 V, short current of ±3 µA, and a maximum power density of 45 mW/m^2^. For the demonstration of applications, the g-C_3_N_4_ doped TENG was fabricated into an energy flag to scavenge wind energy and sensor devices for detecting human motions. 

## Data Availability

Not applicable.

## Supplementary Materials

The following supporting information can be downloaded at: https://www.mdpi.com/article/10.3390/polym14153029/s1, Figure S1: (a) Open-circuit voltage, (b) short circuit current, and (c) charge transfer output of PAN electrospinning membrane TENG with different frequencies of 2 Hz, 3 Hz, 4 Hz, 5 Hz, 6 Hz, 7 Hz, and 10 Hz under the same impact force of 100 N, Figure S2: Electronic output of PVDF electrospinning membrane with and without g-C_3_N_4_ doping, Figure S3: Degradation of different electrospinning membranes under 365 nm ultraviolet for one month, Figure S4: Comparison between common nylon film and electrospinning membrane PA_66_, Figure S5: Test of beat number impact on PAN and PA_66_ membranes, Figure S6: Air resistance of nylon fabric, electrospinning membranes of PA_66_ with and without g-C_3_N_4_ doping, Figure S7: Scanning electron microscope images of (a) electrospinning membrane and (b) graphitic carbon nitride powder at different magnifications, Video S1: Demonstration of lighting 40 LEDs by hand tapping electrospinning membrane TENG.
