# Supplementary material for: Wearable Triboelectric Nanogenerators Based on Polyamide Composites Doped with 2D Graphitic Carbon Nitride
Source: Polymers (Basel). 2022 Jul 26;14(15):3029. doi: 10.3390/polym14153029 (PMC9332467; doi:10.3390/polym14153029)
Supplement: Supplementary file 1 [file polymers-14-03029-s001.zip › Supporting Information.pdf]

Supporting Information

Polyacrylonitrile (PAN) electrospinning membrane was also fabricated and experimented with the same structure and circumstance and fabricated to TENG as a positive dielectric with PTFE films as negative dielectric, then experimented with the electricity with different frequencies under 100 N, and short circuit current increased from about ±1 μA to ±4 μA as frequency from 2 Hz to 10 Hz.

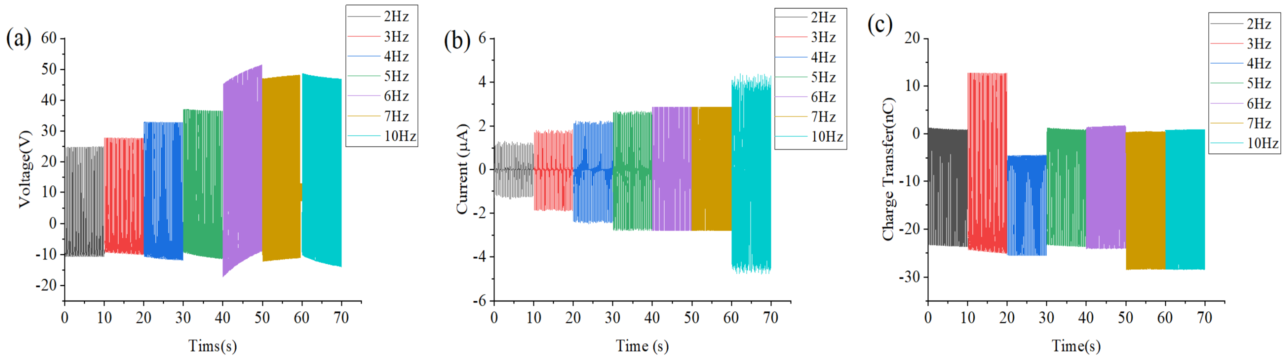

Figure S1. (a) Open-circuit voltage (b) short circuit current (c) charge transfer output of PAN electrospinning membrane TENG with different frequencies 2 Hz, 3 Hz, 4 Hz, 5 Hz, 6 Hz, 7 Hz, 10 Hz under the same impact force of 100 N.

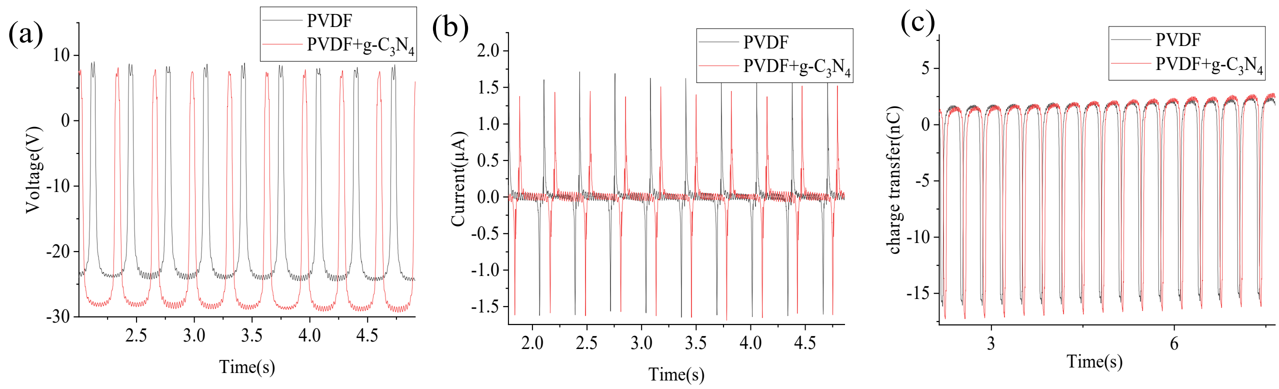

Figure S2. Electronic Output of PVDF electrospinning membrane with and without g-C_3N_4 doping of (a) open-circuit voltage (b) short circuit current (c) charge transfer.

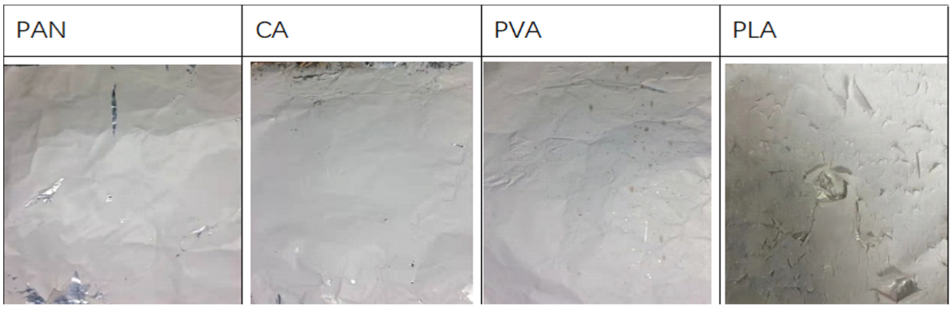

Figure S3. Degradation of different electrospinning membranes under Ultraviolet 365nm for one month

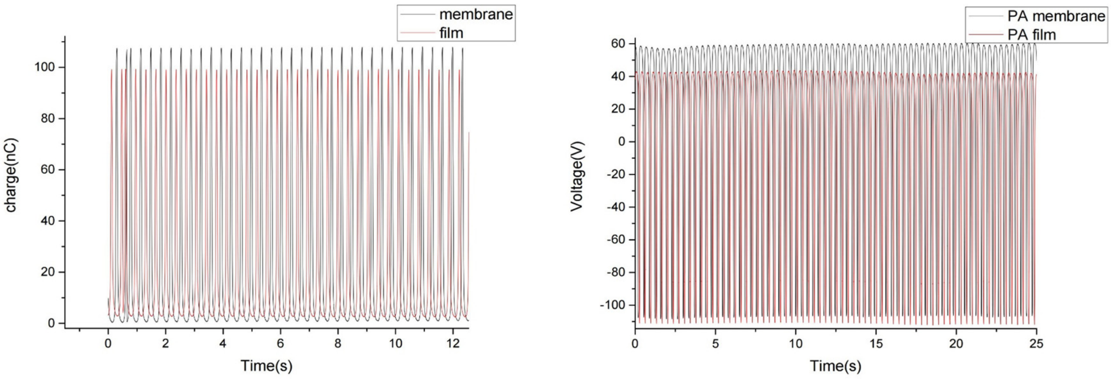

Figure S4. Comparison between common nylon film and electrospinning membrane PA_{66}

**PA_{66}** 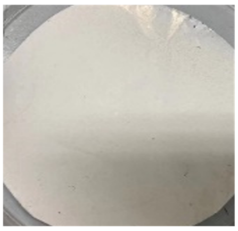	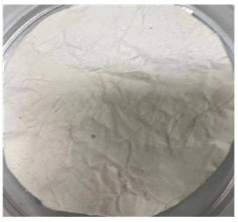	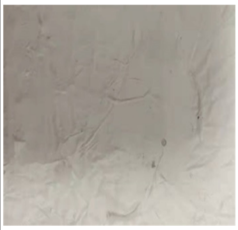	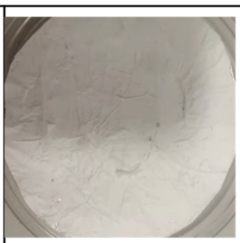
original	5000beat	10000beat	15000beat
**PAN** 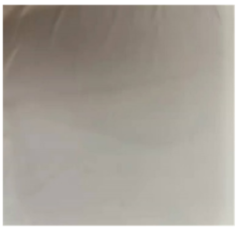	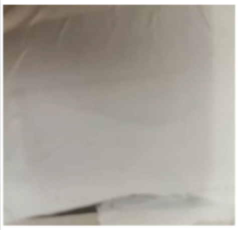	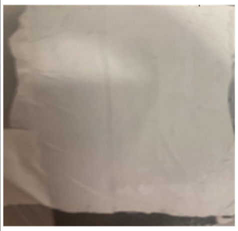	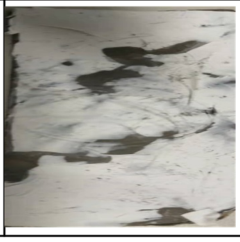

Figure S5. Test of beat number impact on PAN and PA_{66} membranes

Nanofibers from the electrospinning process often show good mechanical strength, which is contributed to the high orientation of polymer molecules in the fibers along the electrospun fiber axis. Softness and flexibility of the electrospun membrane also allow it to fit various conformations including the curvy body shape and the deformation caused by human motions. Furthermore, air permeability of materials is a critical factor in determining wear comfort. The air permeability of commercially available nylon fabric, and electrospinning membranes with and without g-C_3N_4 doping was evaluated by KES-F8-AP1 Air Permeability Tester (KATO TECH CO., LTD.). The resistance demonstrated how difficult the air can pass through the material. It was found that the resistance of electrospun membranes was only slightly higher than that of nylon fabric, indicating the air permeability of electrospun membranes was comparable to apparel fabric. This result could be supported by the high porosity formed between the intersections of electrospun nanofibers, which allowed easy passage of air.

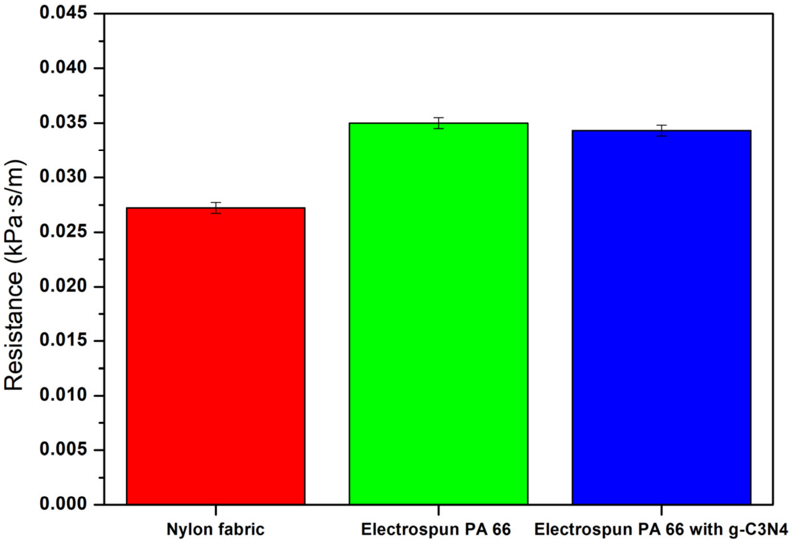

Figure S6. Air resistance of nylon fabric, electrospinning membranes of PA_{66} with and without g-C_3N_4 doping

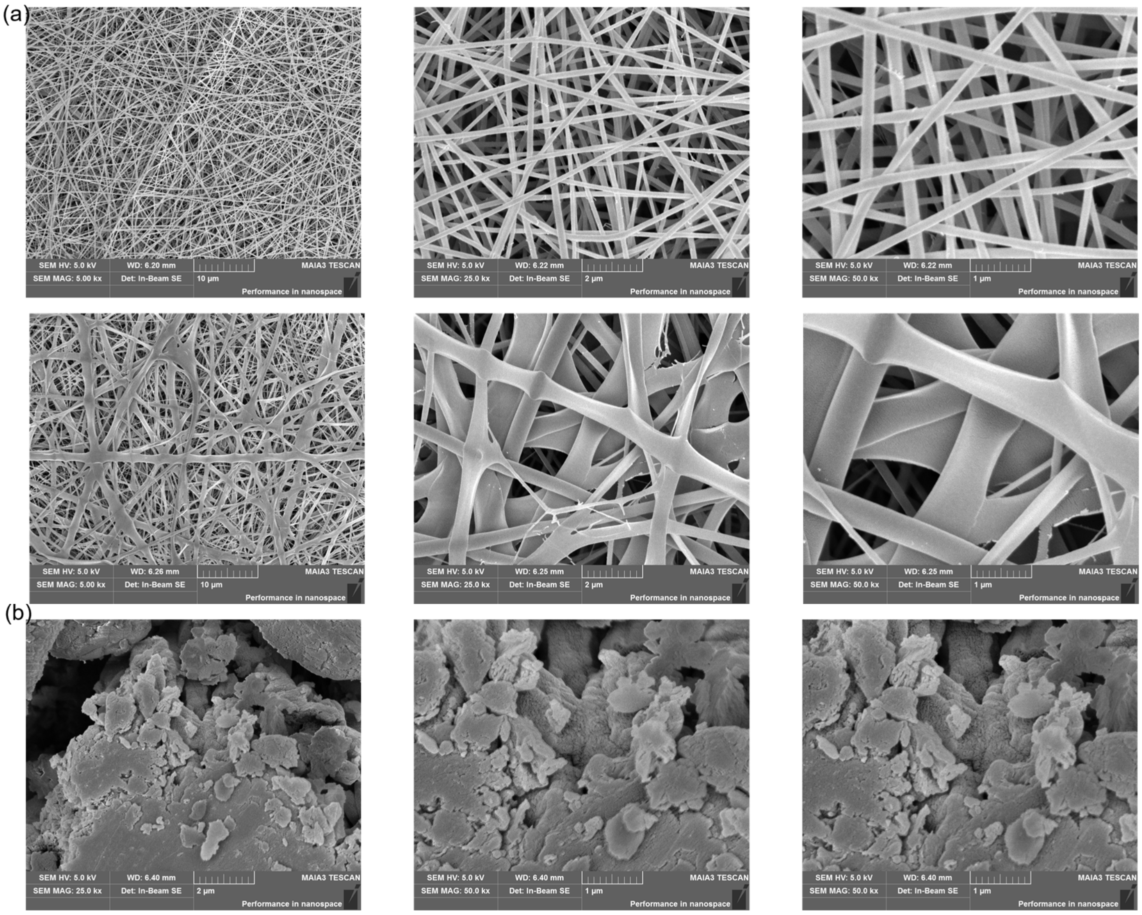

Figure S7. Scanning electron microscope images of (a) electrospinning membrane and (b) graphitic carbon nitride powder at different magnification.

Video S1: Demonstration of lighting 40 LEDs by hand tapping electrospinning membrane TENG
